# Natural IgG against S-Protein and RBD of SARS-CoV-2 Do Not Bind and Hydrolyze DNA and Are Not Autoimmune

**DOI:** 10.3390/ijms232213681

**Published:** 2022-11-08

**Authors:** Anna M. Timofeeva, Sergey E. Sedykh, Evgeny A. Ermakov, Andrey L. Matveev, Eva I. Odegova, Tatiana A. Sedykh, Dmitry N. Shcherbakov, Iuliia A. Merkuleva, Ekaterina A. Volosnikova, Valentina S. Nesmeyanova, Nina V. Tikunova, Georgy A. Nevinsky

**Affiliations:** 1 SB RAS Institute of Chemical Biology and Fundamental Medicine, 630090 Novosibirsk, Russia; 2 Faculty of Natural Sciences, Novosibirsk State University, 630090 Novosibirsk, Russia; 3 State Research Center of Virology and Biotechnology Vector, 630559 Koltsovo, Russia; 4 Department of Physical-Chemistry Biology and Biotechnology, Altay State University, 656049 Barnaul, Russia

**Keywords:** SARS-CoV-2, COVID-19, anti-dsDNA-IgGs, autoimmunity, spike protein, RBD, DNA, coronavirus

## Abstract

Since the onset of the COVID-19 pandemic, numerous publications have appeared describing autoimmune pathologies developing after a coronavirus infection, with several papers reporting autoantibody production during the acute period of the disease. Several viral diseases are known to trigger autoimmune processes, and the appearance of catalytic antibodies with DNase activity is one of the earliest markers of several autoimmune pathologies. Therefore, we analyzed whether IgG antibodies from blood plasma of SARS-CoV-2 patients after recovery could bind and hydrolyze DNA. We analyzed how vaccination of patients with adenovirus Sputnik V vaccine influences the production of abzymes with DNase activity. Four groups were selected for the analysis, each containing 25 patients according to their relative titers of antibodies to S-protein: with high and median titers, vaccinated with Sputnik V with high titers, and a control group of donors with negative titers. The relative titers of antibodies against DNA and the relative DNase activity of IgGs depended very much on the individual patient and the donor, and no significant correlation was found between the relative values of antibodies titers and their DNase activity. Our results indicate that COVID-19 disease and vaccination with adenoviral Sputnik V vaccine do not result in the development or enhancement of strong autoimmune reactions as in the typical autoimmune diseases associated with the production of anti-DNA and DNA hydrolyzing antibodies.

## 1. Introduction

Some viruses are known to trigger autoimmune processes in genetically predisposed people. For example, infection with parvovirus B19 was shown to trigger the production of autoantibodies: antinuclear antibodies, antibodies against double-stranded DNA, and others and activate the development of autoimmune pathologies [1,2]. Moreover, viruses can cause autoantibodies to be produced by antigen-dependent mechanisms, such as molecular mimicry, and antigen-independent means, such as impaired immune tolerance due to inflammation [3].

With some clinical symptoms of COVID-19 corresponding to those of autoimmune diseases, one of the fundamental questions in the COVID-19 pathogenesis is whether infection with SARS-CoV-2 is a risk factor for the autoimmune complication development after the patient recovery. Antibodies to several autoantigens have been found in patients with COVID-19: antibodies to phospholipids, antinuclear antibodies, antibodies to interferons, and others. However, no COVID-19-specific autoantibodies that could serve as a marker for development of autoimmune reactions have been described so far [4]. Several autoimmune diseases were reported following COVID-19, such as acute hemolytic anemia, macrophage activation syndrome, Kawasaki disease, Guillain–Barré syndrome, Miller–Fischer syndrome, and autoimmune thrombotic thrombocytopenic purpura, with a number of autoantibodies detected [5,6,7].

Autoimmune processes in patients recovered from COVID-19 may be due both to the ability of the virus to induce hyperstimulation of the immune system and to the molecular similarity of the virus and host antigens [8]. Extensive damage to the lungs and other organs during coronavirus infection was shown to result in a variety of autoantibodies being produced [9]. Up to now, a number of autoantibodies have been described to be produced in patients with a severe course of COVID-19 [10,11,12]. A lot of these autoantibodies interfere with normal functioning of the immune system and influence the severity and progression of the disease. For example, the production of autoantibodies against annexin A2 and other immunomodulatory proteins was shown to be associated with severe COVID-19 [13,14].

Antinuclear antibodies are considered to be a hallmark of the autoimmune diseases of connective tissue, such as systemic lupus erythematosus (SLE), Sjögren’s syndrome, systemic scleroderma, and idiopathic inflammatory myopathies [15]. Antinuclear antibodies include diverse autoantibodies targeting a variety of intracellular antigens in different cellular compartments, including nucleus components (chromatin, nuclei, and nucleoplasm, and histones), nuclear envelope components, mitotic spindle apparatus, and cytosol [16]. One-third to one-half of patients with severe COVID-19 were estimated to have antibodies against nuclear antigens [17], all studies indicating worse outcomes in patients with positive antinuclear antibodies than in patients without such antibodies. Furthermore, most positive samples were found to contain antibodies against ribonucleoprotein and other nuclear antigens: chromatin, centromere B, SS-A, SS-B cytoplasmic antigens, and double-stranded DNA [11].

In addition, viruses can cause abnormalities in the human immune system, resulting in the production of antibodies with catalytic activity (abzymes) [18,19]. Catalytic antibodies have been found in several viral infections (tick-borne encephalitis, HIV infection [20,21]), bacterial infections [22], and several autoimmune diseases [23,24]. Autoimmune diseases are accompanied by the formation of catalytic antibodies hydrolyzing DNA, RNA, oligonucleotides, proteins, peptides, and oligosaccharides and possessing oxidoreductase activity [18,21,25,26,27,28], and other activities [29,30].

DNA-hydrolyzing antibodies were found in elevated concentrations in the blood of patients with SLE [31,32], multiple sclerosis (MS) [33,34], systemic scleroderma [31], schizophrenia [35,36], spondyloarthritis, polyarthritis [37], HIV infection [25,38], tick-borne encephalitis [39], several bacterial infections [40], and other diseases [29,30]. These antibodies are known to be one of the earliest markers of autoimmune pathologies [26]. IgG with DNase activity is the first statistically significant marker of autoimmune pathology, with this activity detected even in pre-disease stages, e.g., when no visible markers of SLE are present. The relative activity of antibodies with DNA-hydrolyzing activity from patients suffering from various diseases increases in the following order: diabetes < viral hepatitis ≈ tick-borne encephalitis < polyarthritis ≤ Hashimoto’s thyroiditis < HIV/AIDS ≤ MS < SLE [19,35].

In some viral infections, DNA-hydrolyzing antibodies are detected. For example, the blood IgG antibodies of 96% of HIV-infected patients were found to exhibit DNase activity [26]. At the same time, no statistically significant correlation was found between the level of DNA hydrolysis, HIV subtype, viral load, stage of HIV infection, and estimated period of infection [25]. Natural DNA-hydrolyzing antibodies found in the blood of HIV-infected patients can reduce the concentration of extracellular DNA produced by apoptosis or other pathways, thereby decreasing the probability of autoimmune pathologies and having a beneficial effect.

Autoantibodies in patients with COVID-19 were mainly detected during the acute period of infection. However, in order to elucidate a possibility of activation of autoimmune reactions induced by the virus, it is of great interest to test the activity and/or cross-reactivity of antibodies several weeks and months after the COVID-19 infection. In this work, we studied antibodies to the S-protein of COVID-19-infected patients after their recovery and of those vaccinated with Sputnik V to assess the contribution of these antibodies to a possible development of autoimmune reactions. We tried to answer whether antibodies specific to SARS-CoV-2 proteins are cross-reactive in the long term after recovery from COVID-19 or vaccination.

Here, we compared the relative activity of IgG in hydrolysis of DNA in patients recovered from COVID-19 and vaccinated with Sputnik V with those of conditionally healthy donors. Given the physicochemical properties of the S-protein and its receptor binding domain (RBD), we tested the hypothesis that antibodies to S-protein and/or RBD could be cross-reactive and bind and/or hydrolyze DNA, which might be a predisposing factor for autoimmune reactions and multiorgan lesions following SARS-CoV-2 infection. Polyclonal antibody preparations specific to SARS-CoV-2 proteins were obtained from the blood of COVID-19 and/or Sputnik V vaccinated donors. We have determined the ratio of IgG1-IgG4 subclasses and characterized the DNase activity of IgG preparations compared with subfractions against RBD and S-protein.

## 2. Results

### 2.1. Antibodies to S- and N-Proteins of SARS-CoV-2

Blood plasma samples were obtained from group of patients (further patients are named donors recovered of COVID-19 or healthy donors vaccinated by Sputnik V) and control group of conditionally healthy donors, which have no clinical symptoms of any autoimmune or viral diseases in Novosibirsk between October 2020 and May 2021. By collecting samples during this time period, we traced the first contact with the SARS-CoV-2 virus and selected vaccinated donors who had not previously encountered the virus. We conducted IgG screening for the S- and N-proteins of the SARS-CoV-2 virus by enzyme-linked immunosorbent assay (ELISA). After COVID-19 infection, antibodies against the viral surface protein (S) and nucleocapsid (N) were detected in plasma. The adenovirus vaccine Sputnik V contains the S-protein gene of SARS-CoV-2, resulting in the production of antibodies only against the S-protein, with no antibodies against the N-protein [41]. Thus, ELISA analysis of IgG against S- and N-protein of SARS-CoV-2 allowed dividing patients and donors into groups according to the PR of IgG to S-protein and excluding asymptomatic cases from the vaccinated and unvaccinated donor groups.

Four groups of 25 patients and donors each were used in current analysis. Among more than 500 patients recovered from COVID-19, only 50 were selected for this study and divided into two groups according to their antibody titer to S-protein: (1) HTD—high titer of S-protein patients who underwent COVID-19 and had a high antibody titer, positivity rate (PR) > 7 (corresponding to titer 1:641); (2) MTD—recovered from COVID-19 with median titer of S-protein: (3) HTV—patients with high titer to S-protein, who did not have COVID-19 and were vaccinated with Sputnik V; (4) NTD—group of apparently healthy donors demonstrating negative titer to S-protein, who had no COVID-19, and were not vaccinated. The HTD and MTD groups included patients with positive OT-PCR and clear signs of SARS-CoV-2 infection: high fever, anosmia, and ageusia. HTV and NTD groups included only patients and donors who were seronegative for N-protein. For this analysis, we selected only plasma from donors who were negative for autoimmune processes and chronic infections affecting the production of anti-DNA IgG and catalytic antibodies (e.g., HIV infection).

The HTD group included patients with high titers of SARS-CoV-2 virus S-protein antibodies: 40% male (10 individuals), the mean age was 45.5 ± 16.3 years, the mean time between the onset of the first symptoms and blood collection was 12 ± 6 weeks, the mean PR value of IgG against S-protein was 10.3 ± 4.1. The MTD group included patients with medium antibody titer to SARS-CoV-2: 24% male (6 individuals), the mean age of 40.4 ± 10.9 years, the mean time between the onset of the first symptoms and blood collection was 11.0 ± 5.0 weeks, the mean PR value was 5.1 ± 1.5. The HTV group included patients vaccinated with two doses of Sputnik V who had no antibodies to N-protein coronavirus and a high titer of antibodies against S-protein: 40% male (10 individuals), the mean age was 42.8 ± 13.0 years, the mean time between first vaccination and blood collection was 7.0 ± 2.0 weeks, the mean PR value was 10.0 ± 3.7. The NTD control group included unvaccinated conditionally healthy donors without N- and S-protein antigens: 28% male (7 individuals), the mean age was 41.0 ± 10.2 years, and the mean PR was 0.4 ± 0.2. Shown in Table 1 are patients and donors characteristics and their statistical treatment.

Using Pearson’s chi-squared test, it has been shown that sex and age ranges did not differ significantly across the three groups (see Table 1). Significant differences in the average time between the first symptoms and blood sampling (df = 2; F = 6.65; *p* = 0.002), as well as the average PR (df = 3; F = 246; *p* = 6.3 × 10^−45^) were revealed among the four groups using One Way ANOVA. The Tukey post hoc test showed that the mean time between the first symptoms and blood sampling in the HTV group differed significantly from the HTD and MTD groups. The mean PR in the MTD group were significantly different from HTD and HTV groups. Moreover, the mean PR in the NTD group were significantly different from the other three groups (Table 1).

### 2.2. IgG Isolation and Characterization

**Figure 1:**
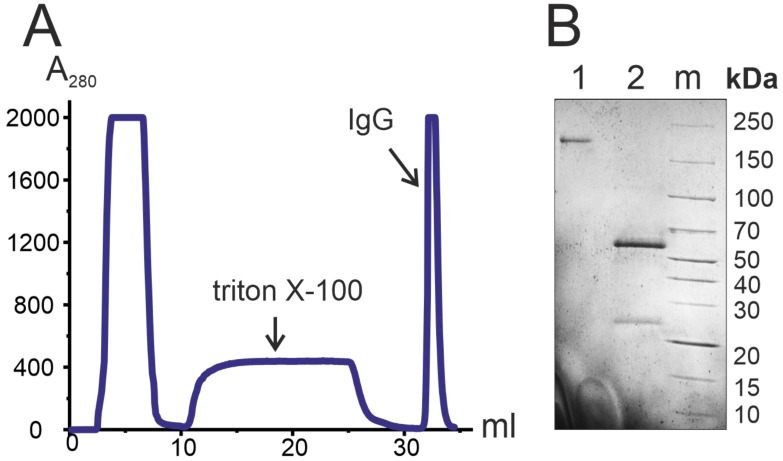
Isolation and electrophoretic analysis of IgG preparation. Profile of IgG isolation from blood plasma of one patient using affinity chromatography on Protein-G-Sepharose (**A**). SDS-PAGE homogeneity analysis of one of the IgG preparations in 4–18% gradient gel (**B**): 1—Intact IgG preparation; 2—IgG boiled with 40 mM DTT; m—protein molecular weight marker (Thermo Scientific, Waltham, MA, USA).

Previously, we published the protocols allowing electrophoretically homogeneous antibody preparations to be isolated from various biological fluids that do not contain any proteins or canonical enzymes [42,43,44]. In this work, we used this protocol to isolate 100 individual IgG preparations of three groups of patients and one group of donors using affinity chromatography of plasma proteins on Protein-G-Sepharose under conditions of nonspecific interactions breakdown. Figure 1A illustrates the affinity chromatography profile of IgG from one of the patients.

**Figure 2:**
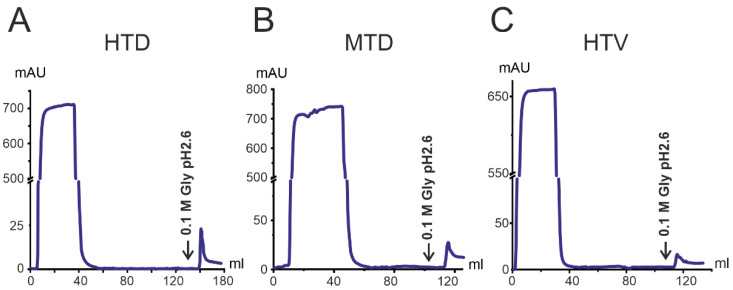
Isolation of anti-RBD-IgG by affinity chromatography of total IgGs on RBD-Sepharose: (**A**) HTD, (**B**) MTD, and (**C**) HTV. Antibodies with different affinities for RBD were eluted with 0.1 M glycine pH 2.6.

Electrophoretic homogeneity of all IgG preparations was shown by SDS-PAGE, with only one band detected above 150 kDa in a region typical for IgG (Figure 1B–1). When IgG was boiled with dithiothreitol (DTT) in the conditions for complete disulfide bond recovery, only two bands were detected (see Figure 1B–2): 50 kDa, H-chain and 25 kDa, L-chain. With no additional bands detected, the resulting IgG preparations were electrophoretically homogeneous. Figure 1B illustrates the analysis of one of the resulting IgG preparations.

Affinity chromatography on Sepharose with immobilized RBD allows obtaining sub-fractions of antibodies, differing in their affinity for these proteins [23,24,45]. Autoimmune pathologies cause the formation of various autoantibodies that can be isolated on appropriate affinity sorbents. For example, in previous works, we obtained antibodies to the myelin basic protein from the blood plasma of MS [23,45], SLE [24,46], and HIV-infection [47] anti-histone antibodies of MS [45,47], and HIV-infected patients [48]. In this work, recombinant RBD and SARS-CoV-2 S-protein were covalently immobilized on CNBr-activated Sepharose. These sorbents were used to isolate specific antibodies to these two antigens. In the NTD group, the donors did not suffer from SARS-CoV-2, so the antibodies in this group contain no specific fraction of anti-RBD- and anti-S-IgG.

Unfortunately, we failed to isolate preparations of antibodies of individual patients with affinity to RBD, as the content of these antibodies in the total IgG pool was too low (2–8 mL of blood was collected from each patient). Therefore, we pooled 1 mg of antibodies from each of the 25 patients of the three groups described above and isolated total antibody preparations against RBD and S-protein. 

From the total pool of IgG from 25 patients, the subfractions with affinity to RBD (anti-RBD-IgGs) were isolated (Figure 2). The analysis of the chromatography profile revealed that the anti-RBD-IgGs content in the plasma of patients recovered from COVID-19 and of vaccinated ones against the SARS-CoV-2 virus was only 1.1–1.4% (0.28–0.34 mg RBD-IgGs were isolated). It is worth noting that the differences in anti-RBD-IgGs between the three patient groups were not significant.

**Figure 3:**
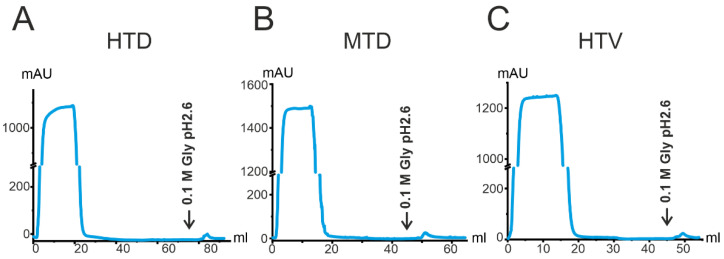
Isolation of S*-IgG by affinity chromatography on S-Sepharose from the IgG antibody preparation, after chromatography on RBD-Sepharose. Patient groups: (**A**) HTD, (**B**) MTD, (**C**) HTV. Elution of antibodies was performed with 0.1 M Gly-HCl pH 2.6.

Following the chromatography on RBD-Sepharose, the fraction with no affinity to the sorbent (fractions corresponding to 0–45 mL, Figure 2) was applied to Sepharose with immobilized S-protein (S-Sepharose). Elution with acidic buffer allowed obtaining antibodies with affinity to other S-protein domains than RBD (S*-IgG). The amount of such S*-IgG was only 0.2–0.6% (0.05–0.15 mg S*-IgGs), shown in Figure 3. Notably, the low amount of S*-IgG is characteristic of both vaccinated and COVID-19 recovered patients.

Our results of IgG chromatography on RBD-Sepharose and subsequent chromatography on S-Sepharose indicate that most antibodies against the SARS-CoV-2 protein after cure from COVID-19 and after vaccination with Sputnik V are RBD-IgG.

### 2.3. Characterization of IgG Subclasses in Blood Plasma

It is known that there are four subclasses of IgG in humans, with more than 90% similar sequences but different functions. For example, the IgG antibody response to a bacterial infection is primarily associated with IgG2, whereas viral infections commonly induce the production of IgG1 and IgG3 [49]. IgG4 is produced during chronic antigen immunization and undergoes HL-fragment exchange in the blood [50]. The relative amount of IgG of different classes and subclasses was found to vary significantly in the blood sera of healthy donors: IgG1 (34–87%), IgG2 (5–56%), IgG3 (0.5–12%), and и IgG4 (7–12%) [51].

**Figure 4:**
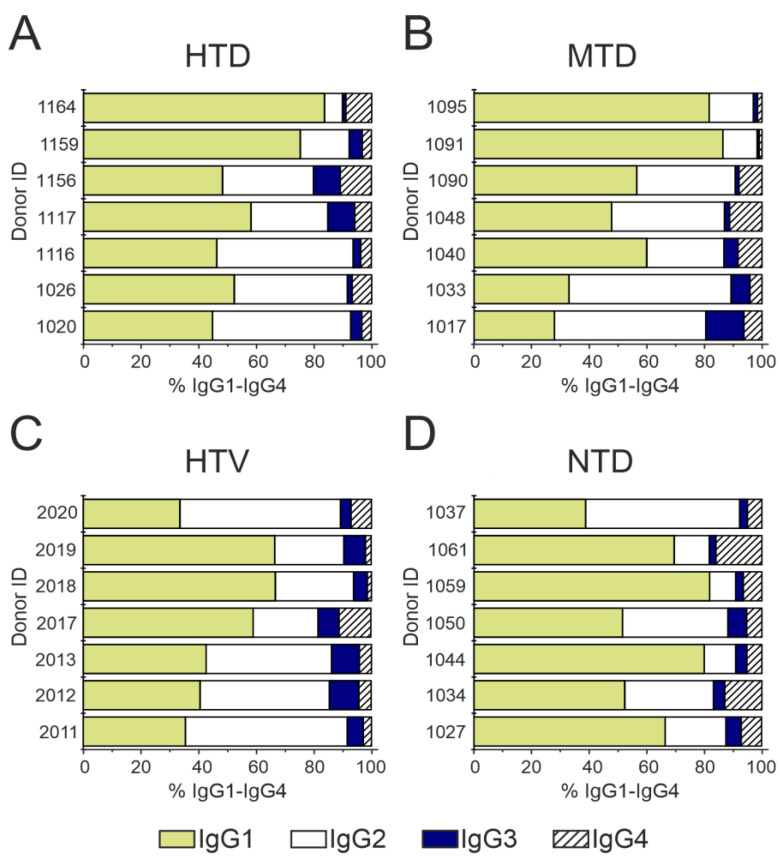
ELISA analysis of IgG1–IgG4 subclasses in the blood plasma of HTD (**A**), MTD (**B**), HTD patients (**C**), and NTD donors (**D**). The measurement error did not exceed 5%.

In this work, we analyzed the ratio of IgG subclasses for seven patients from each of the four groups HTD (Figure 4A), MTD (Figure 4B), HTD (Figure 4C), and NTD (Figure 4D). Consistent with previous results [32,51], we have demonstrated the ratio between IgG subclasses to be unique for each patient or donor and unaffected remarkably by COVID-19 or vaccination. Most patients or donors had a predominant IgG1 subclass (68% of patients and donors from all groups), with several patients, in contrast, having an IgG2 predominance. The relative amount of IgG1–IgG4 determined in this study is generally consistent with the literature data [51]. The Mann–Whitney U Test did not reveal any significant differences in IgG1–IgG4 contents in the four groups under study (*p* > 0.05). The raw data are presented in Table S1.

**Figure 5:**
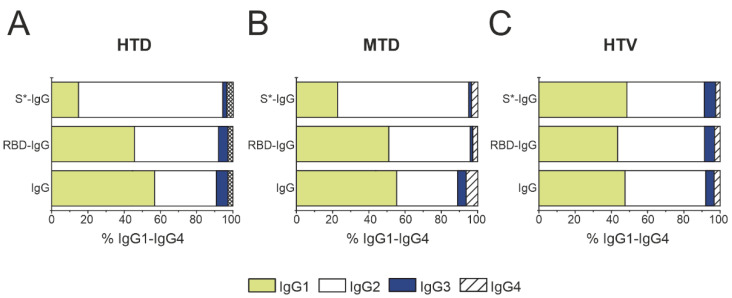
ELISA analysis of IgG subclasses in S*-IgG, RBD-IgG, and IgG preparations with no affinity for RBD- and S-Sepharose in three patient groups: HTD (**A**), MTD (**B**), and HTV (**C**). The measurement error did not exceed 5%.

The contribution of each subclass was determined for RBD-IgG and S*-IgG, with the data shown in Figure 5 and the raw data given in Table S2. Figure 5 demonstrates that IgG1 and IgG3 binding RBD- and S-proteins dominate among the IgG subclasses. This finding is consistent with the fact that these subclasses are associated with recovery and survival in COVID-19 serological studies [52]. It should be noted that the literature data on the analysis of IgG subclasses were obtained during acute SARS-CoV-2 infection, with IgG1 and IgG3 being the dominant IgG subclasses [53]. In this work, we studied the antibodies circulating in the body for several weeks after recovery, with the results describing the condition of the patient who recovered from the disease. The patients chosen for this study had moderate COVID-19, with at least six weeks passing from the onset of the first symptoms until blood sampling.

The distribution of RBD-IgG subclasses correlates with the total preparations: IgG1 ≈ IgG2 > IgG3 > IgG4. Of interest are the results on the subclass contents in the S*-IgG preparations, in both the high and medium titer in COVID-19 patients, IgG2 were significantly predominated: IgG2 >> IgG1 > IgG4 > IgG3.

In summary, the results for the IgG subclass contents in patient serum preparations and in preparations of IgGs and various subfractions indicated no significant differences from the literature data on the subclass contents in the blood of healthy donors [51] and with HIV infection [25]. The data are provided in Table 2.

### 2.4. DNase Activity Assay

Total polyclonal IgG preparations were tested for the presence of DNase activity. Catalytic antibodies with DNase activity are described in the blood of patients with autoimmune (SLE, MS) and viral (HIV infection, tick-borne encephalitis) diseases [54,55]. The relative activities of such IgG with DNase activity are usually relatively low at the onset of mentioned diseases and increase strongly during the development of these pathologies, which leads to pathological reactions [25,26]. It should be noted that antibodies from conditionally healthy donors are usually absent or possess very low catalytic activity in the DNA hydrolysis [18].

**Figure 6:**
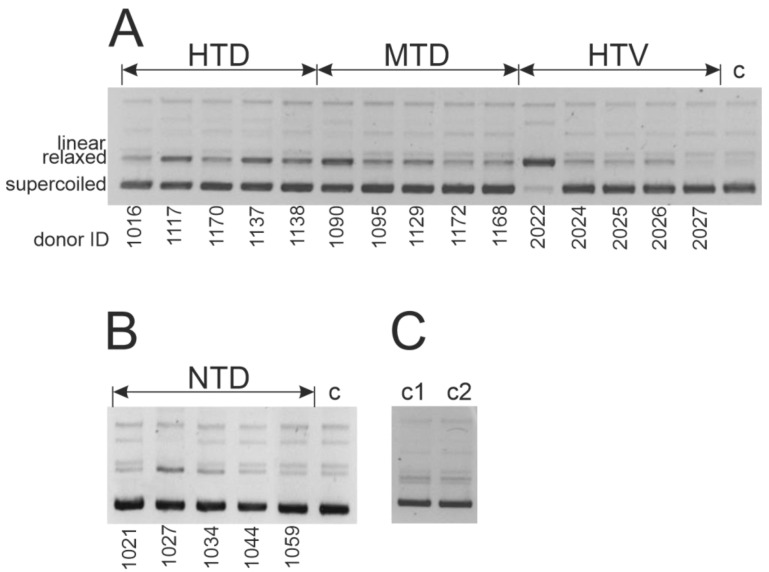
Analysis of the DNA-hydrolyzing activity of IgG preparations. To illustrate, the data for five samples of each group are provided: HTD, MTD, HTD (**A**), and NTD (**B**) in the cleavage of double-stranded supercoiled pBluescript plasmid DNA leading to the relaxed plasmid formation. (**C**)—control samples (c, c1, c2) did not contain IgG samples and were incubated for 48 h (c), 72 h (c1), and 120 h (c2), respectively.

In the current paper, we analyzed DNase activity of three groups of patients (HTD, MTD, and HTV) and apparently healthy donors (NTD). Figure 6 demonstrates the results of the analysis of the supercoiled plasmid DNA hydrolysis products by agarose gel electrophoresis for IgG preparations isolated from the blood of HTD, MTD, and HTV (Figure 6A), and NTD (Figure 6B). The DNase activity level was measured by the conversion of the supercoiled form of DNA into a linear and relaxed form due to the formation of single- or double-stranded breaks in the plasmid DNA.

**Figure 7:**
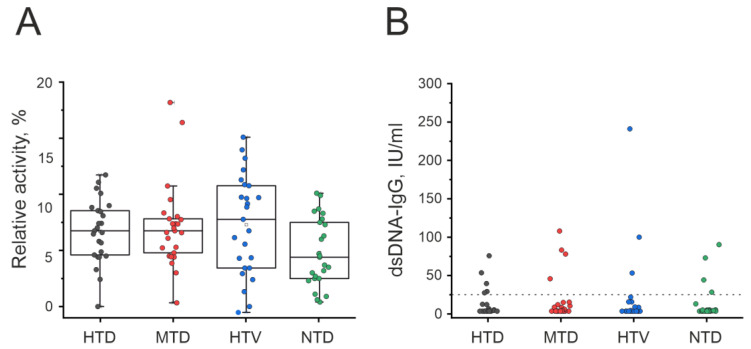
Summarized data of the DNAse activity in HTD, MTD, HTV, and NTD: 100% was taken as complete hydrolysis of plasmid in 24 h by antibodies in a concentration of 0.01 mg/mL (**A**). Contents of anti-dsDNA-IgG shown no statistically significant differences between groups (**B**).

The Figure 7A summarizes the DNA-hydrolyzing activity data for the three patient groups and one conditionally healthy donors group under study. It should be noted that none of the patients and donors had a history of autoimmune pathologies or viral diseases leading to the development of autoimmune pathologies, such as HIV infection or other chronic or persistent viral diseases.

All 25 healthy donors of the control NTD group showed low DNase activity (0.4–10.1%, average value 4.8 ± 3.0%) (Table S3). The relative values of DNase activity in the three groups of patients analyzed varied greatly depending on the patient (from 0 to 18.2%). Interestingly, the average values of DNase activity in three patient groups were ~1.5-fold higher than for the control group of apparently healthy donors: HTD (6.7 ± 2.8%, *p* = 0.03), MTD (7.1 ± 3.8%, *p* = 0.04), HTV (7.3 ± 4.4%, *p* = 0.04), these differences were statistically significant. At the same time, no statistically significant difference was found in the relative values of DNase activity between the three groups of patients (HTD, MTD, and HTV), *p* = 0.6–0.9. Since it is practically impossible to diagnose autoimmune diseases at the earliest stages, when analyzing these data, it should be emphasized that the donors of the control group should be considered conditionally healthy people who do not have obvious indicators of any autoimmune or viral diseases.

ELISA was used to analyze the presence of antibodies against dsDNA in individual blood plasma preparations of the analyzed groups. According to the manufacturer’s instructions, an antibody titer of less than 25 IU/mL was considered negative. However, anti-dsDNA-IgG titers were not negative for 20%, 16%, 16%, and 16% of patients in the HTD, MTD, HTD, and donors in NTD group, respectively (Figure 7B). It should be noted that the sets of relative concentrations of anti-DNA-IgGs were different in each of the four groups and did not correspond to the Gaussian distribution. The average values for each of the groups are (IU/mL): HTD (10.9 ± 21.8), MTD (16.6 ± 34.0), HTV (20.7 ± 59.4), NTD (11.3 ± 26.8) see Table S3. The average values of anti-DNA-IgG for two groups of patients (MTD and HTV) were higher than the values for the group of conditionally healthy donors in 1.5–1.8 times. At the same time, no statistically significant difference in anti-DNA-IgG content was found for all four groups, *p* values are varied from 0.43 to 0.93.

We have evaluated the correlation between anti-dsDNA-IgG titers detected by ELISA and DNA-hydrolyzing activity levels in the analyzed groups, with Spearman’s rank correlation test showing no significant correlations in any group between anti-dsDNA-IgGs and DNA-hydrolyzing activity levels: r1 = 0.27 (HTD), r2 = 0.02 (MTD), r3 = 0.10 (HTV), and r4 = 0.24 (NTD). In all studied groups, the *p*-value was >0.05. The raw data are presented in Table S3.

Canonical enzymes, such as DNase I, possess high catalytic activity, while catalytic IgG possesses much lower activities. Table S4 presents the results of concentration-dependent activity analysis of canonical DNase I. The commercial DNase I preparation was used as a positive control (see Figure S1), demonstrating that depending on the patient ID, specific DNase activity of IgG varies from 10^−19^ to 10^−15^ U to one mg of IgGs analyzed.

Catalytic antibodies, including DNA-hydrolyzing antibodies, are markers for autoimmune pathologies such as SLE and MS [27,45,56]. Catalytic antibodies are absent or significantly less active in conditionally healthy donors than in case of autoimmune or viral pathology [26,30]. The IgG preparations analyzed in this study demonstrated low DNase activity in the case of three groups of patients (HTD, MTD, and HTV) having no typical clinical symptoms of autoimmune disorders at the time of blood collection. Average IgGs’ DNase activity in these three groups was only 1.4–1.5 times higher compared to conditionally healthy donors, but several orders lower compared to the patients with pronounced autoimmune diseases [19,29]. This indicates that patients recovered from the SARS-CoV-2 have no strong changes in the immune system leading to the development of autoimmune diseases associated with the production of DNA-hydrolyzing antibodies.

**Figure 8:**
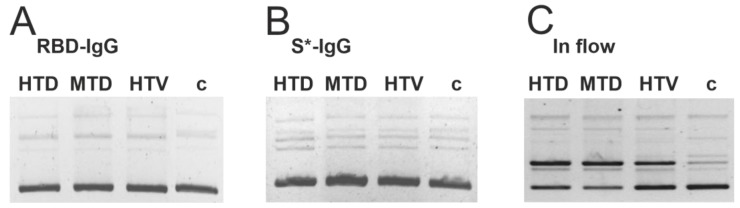
The DNA-hydrolyzing activity of anti-RBD-IgG (**A**) and S*-IgG (**B**) of HTD, MTD, and HTV groups. As a control, the activity of the pooled IgG preparation from 25 patients after anti-RBD-IgG and S*-IgG isolation is shown (**C**). The activity was evaluated in the cleavage of double-stranded super-stranded (sc) plasmid DNA pBluescript. The c-track corresponds to supercoiled plasmid DNA incubated without antibodies.

We have analyzed the DNase activity of anti-RBD-IgG and anti-S*-IgGs from the three patient groups (Figure 8A,B). RBD-IgG and S*-IgG from both vaccinated patients and those who recovered from the disease did not exhibit detectable DNase activity. It should be noted that IgGs without affinity for S-protein demonstrated DNA hydrolysis (Figure 8C). Since anti-DNA-IgG without affinity for S-protein should be eluted when applied to the affinity sorbent, it can be assumed that DNase activity of IgGs is not associated with antibodies possessing affinity to viral proteins formed during vaccination or COVID-19 infection. 

DNase activity is known to be one of the earliest symptoms of SLE and MS [57]. Our data indicate that within three months after the illness, the patients tested had no clinical (vitiligo, rash, restricted movement, joint pain, or signs of autoimmune organ or tissue damage) or significant biochemical symptoms of autoimmune pathology. Almost two years after blood collection, none of the patients have reported any substantial clinical symptoms of autoimmune reactions. As soon as the pandemic is over, we are to perform another screening of anti-DNA and DNA-hydrolyzing antibodies in the blood of the same patients to analyze the development of possible autoimmune pathologies.

## 3. Discussion

In this work, we investigated whether COVID-19 is a risk factor for the development of autoimmune complications associated with production of anti-DNA and DNA-hydrolyzing IgGs. Such antibodies are characteristic of autoimmune pathologies such as SLE and MS. A number of immune processes have been described in COVID-19, including cytokine storms [58], antibodies to interferons [59], antiphospholipid antibodies: anti-cardiolipin, anti-β2 glycoprotein, and anti-phosphatidylserine/prothrombin [10]. To the best of our knowledge, the appearance of anti-DNA and DNA-hydrolyzing antibodies in the blood of COVID-19 patients was not studied yet. 

A significant part of our work was analyzing the production of the antibodies concerned in vaccinated patients (both those having recovered from COVID-19 and those who had never had it before). We have examined the production of anti-DNA and DNA-hydrolyzing antibodies in patients vaccinated with the adenovirus vaccine Sputnik-V, an analog of Oxford-AstraZeneca, Convidecia, Janssen, and similar vaccines. The production of autoantibodies might be a highly undesirable post-vaccination consequence.

It has been shown that the average relative DNase activity of IgGs from the blood of three groups of patients is 1.4–1.5 times higher (*p* < 0.05) comparing to the conditionally healthy donors (Table S3). Spontaneous development of SLE in SLE-prone MRL-lpr/lpr mice [60] and EAE in EAE-prone C57BL/6, Th, and 2D2 mice spontaneously developing this disease [61,62,63] are caused by changes in the differentiation of bone marrow stem cells, leading to the production of B-lymphocytes. The antibodies, produced with these cells are harmful for the organism since they hydrolyze proteins, peptides, DNA, and RNA. Immunization of MRL-lpr/lpr, C57BL/6, Th, and 2D2 mice with DNA and its complexes, with proteins, peptides leads to the development of autoimmune pathology associated with changes in bone marrow stem cells proliferation and catalytic antibody production [64]. Unlike in the case of mice predisposed to autoimmune reactions, or immunization of other mouse lines, this is not associated with changes in the differentiation of bone marrow stem cells. Immunization of rabbits with protein complexes with DNA or RNA leads to the production of catalytic antibodies hydrolyzing both DNA and RNA [61,65,66]. Since COVID-19 is associated with the appearance of virus components in the patients’ blood, including ribonucleoprotein complexes, this may result in some slight increase in DNase activity of natural IgG, not lead by changes in differentiation of bone marrow stem cells.

Our results suggest that anti-DNA-IgG and DNA-hydrolyzing IgG in COVID-19 patients are unlikely associated with the changes in differentiation of stem cells of infected patients and vaccination does not lead to the development of autoimmune processes.

Although a variety of autoantibodies have been described in COVID-19, it should be noted that most studies focus on antibodies isolated from patients’ blood during the acute phase of infection. However, we believe that our analysis of autoantibodies after recovery from COVID-19 have a critical importance. The emergence of anti-DNA-IgG, DNA-hydrolyzing antibodies and other autoantibodies in recovered patients could reflect immune activation during infection or early loss of tolerance, which could possibly lead to chronic autoimmune pathology [18,29].

In this study, we have demonstrated that there are no statistically significant differences in DNase activity between the groups of patients recovered from COVID-19 and patients vaccinated with Sputnik V.

Most of the literature focusing on the analysis of humoral immunity in COVID-19 addresses antibodies with diagnostic importance: against the S-protein, and its RBD fragment, and N-protein. Since RBD is the target of many neutralizing antibodies against SARS-CoV [67], anti-RBD-IgG are particularly interesting objects of investigation. Unfortunately, the isolation of anti-RBD-IgG individually for each patient was not possible due to their low content: even in the group of patients with a high titer of antibodies to SARS-CoV-2 registered by the ELISA, such antibodies accounted for no more than 1.1–1.4% of the total pool.

The canonical ratio of IgG1–4 subclasses in the blood of healthy donors is 67% for IgG1, 22% for IgG2, 7% for IgG3, and 4% for IgG4. Here, we show that the ratio of subclasses in total IgG preparations and RBD-IgG preparations were comparable. However, the S*-IgG of COVID-19 patients differed markedly from other preparations in the ratio of IgG subclasses. For example, IgG2 significantly predominated in the HTD and MTD groups of patients. IgG1 and IgG2 predominated in patients vaccinated with Sputnik V, but it is difficult to draw unequivocal conclusions about the physiological significance of these results. However, we believe that they provide further evidence that the full-length S-protein rather than its RBD fragment should be used for vaccination and in ELISA.

Thus, our results indicate that COVID-19 and vaccination with the adenovirus vaccine Sputnik V do not result in the development or enhancement of autoimmune pathology associated with the production of anti-DNA and DNA-hydrolyzing antibodies.

## 4. Materials and Methods

### 4.1. Donors and Patients

The study was approved by the Local Ethics Committee of the Institute of Chemical Biology and Fundamental Medicine (Protocol Number 21-4 from 7 August 2020), including the written consent of patients and healthy donors to present their blood for scientific purposes (according to guidelines of the Helsinki ethics committee).

Vacuum tubes with anti-coagulation compound (EDTA) were used to collect fasting venous blood. Blood tubes were centrifuged at 3000× *g* for 15 min in a 5810 centrifuge (Eppendorf, Hamburg, Germany). Plasma separated from the red cell mass was divided into aliquots and stored at −70 °C.

For this study, plasma samples of 100 volunteers were selected from a collection of samples obtained from patients of different ages with a different course of COVID-19 disease: 50 patients who recovered from COVID-19 (NTD, MTD); 25 patients vaccinated with two doses of Sputnik V (HTV) with high antibody titer; 25 donors who had no COVID-19 and no vaccinated against SARS-CoV-2 (NTD). COVID-19 in all donors was confirmed by PCR and ELISA against S- and N-protein of SARS-CoV-2. The presence of antibodies against S-protein and absence of antibodies against N-protein in HTV patients was also analyzed by ELISA.

The samples were collected in Novosibirsk between October 2020 and May 2021. Consequently, these samples display the B-lymphocyte antigenic response to the Wuhan strain. 

### 4.2. ELISA of Antibodies

The IgG content against S-protein and N-protein SARS-CoV-2 was evaluated using the “Antigma G” ELISA system (Generium, Volginskiy, Russia) according to the manufacturer’s instructions. After determining the antibody titer to S-protein, patients and donors were divided into four groups depending on the content of antibodies to S-protein: with high titer of anti-S-IgG and PR above 7; with medium titer of anti-S-IgG and PR from 4 to 7; with low titer of anti-S-IgG and PR from 1 to 4; and negative samples with PR titer <1. Enzyme immunoassay for N-protein antibodies was used to confirm the absence or presence of a history of COVID-19. Four groups were formed: HTD—Patients who recovered from COVID-19 with high titer of anti-S-IgG, MTD—Patients who recovered from COVID-19 with medium titer of anti-S-IgG, HTV—Patients vaccinated with two doses of Sputnik V with high titer of anti-S-IgG, NTD—Donors who had no COVID-19 and demonstrating negative titer to S- and N-protein.

### 4.3. Antibody Purification and Analysis

Electrophoretically and immunologically homogeneous IgG from the blood plasma of each patient was obtained by affinity chromatography on Protein-G-Sepharose (GE Life Sciences, New York, NY, USA), similarly to our previous papers [24,35,68]. Electrophoretic homogeneity of the IgG preparations was shown using a 4–15% gradient SDS-PAGE followed by Coomassie blue staining. Each IgG preparation was analyzed in the presence and absence of 40 mM DTT (boiled with DTT for 2 min).

### 4.4. Isolation of Antibodies against S-Protein and RBD

The RBD fragment of S-protein was prepared in a CHO-K1 cell line containing the RBD coding fragment of the S-protein coding part of the Wuhan-1 SARS-CoV-2 strain (GenBank: MN908947) with codon-optimization for expression in mammalian cells as in [69,70,71]. The synthesized RBD sequence was cloned into the pVEAL2 transposon plasmid in frame with the N-terminal spike signal sequence (MFVFLVLLPLVSSQC) and the C-terminal 10×His-tag. CHO-K1 cells were transfected with the pVEAL2-S-RBD and helper plasmid pCMV (CAT) T7-SB100, encoding SB100 transposase, using Lipofectamine 3000 (Invitrogen, Carlsbad, CA, USA). The transfected cells were selected with puromycin (10 µg/mL), and high-producing clones were obtained by dilution cloning and cultured in roller bottles at 37 °C on DMEM/F-12 (1:1) medium supplemented with 2% FBS and 50 µg/mL gentamicin. RBD expressed in the CHO-K1 culture medium was purified by Ni-NTA and ion exchange chromatography. The RBD samples were dialyzed against PBS and sterilized by 0.22 μm filters.

The pOptiVec_pCAG-S plasmid was used for transient production of the SARS-CoV-2 S-protein. CHO-S cells were cultured at 37°C in a CO_2_ incubator in CD OptiCHO medium (Biolot, St. Petersburg, Russia) in 125 mL Erlenmeyer flasks on a Celltron device (Infors HT, UK) at 160 rpm. Cells were passaged 3–4 times before transfection and seeded in 18 mL of OptiCHO medium containing 6 mM glutamine (Biolot, St. Petersburg, Russia) at a density of 0.5–0.7×10^6^ cells/mL. Cell transfection was performed using PEIpro (Polyplus, France) according to the manufacturer instructions. During transfection, 0.2 mL of PEIpro reagent was mixed with 1 mL of Opti-MEM medium. The resulting mixture was incubated at room temperature for 2 min. Aliquote of 0.1 mg of plasmid DNA was dissolved in 1 mL of Opti-MEM medium. Both solutions were mixed and incubated for 5 min at room temperature. CHO-S cells were added to the resulting suspension. The medium was changed once a day, 5 times. The recombinant SARS-CoV-2 S protein was isolated from the obtained supernatant.

The sorbents containing immobilized RBD and S-protein were prepared according to the standard protocol using CNBr-Sepharose (GE Life Sciences, New York, NY, USA) and our previously published works [23,72].

IgG was subjected to sequential chromatographic fractionation on columns with immobilized RBD- and S-proteins (RBD-Sepharose and S-Sepharose). The IgG product (1 mg of IgG from each patient, 25 mg of antibodies from each group in total) was applied to a 3 mL of RBD-Sepharose resin pre-equilibrated with 50 mM Tris-HCl pH 7.5, containing 0.15 M NaCl (TBS). The protein yield was monitored by changing the optical density of the eluate at λ = 280 nm. A 1.5 mL fraction was collected and dialyzed against 20 mM Tris-HCl pH 7.5, and the fraction eluted with acidic buffer was neutralized by adding 1/10 by volume of 1 M Tris-HCl pH 8.8. Chromatography was performed on an Akta Start chromatograph (GE Life Sciences, New York, NY, USA), and chromatograms were processed using Unicorn 1.0 software.

The IgG preparation not bound to RBD-Sepharose was then applied to a 10 mL S-Sepharose column pre-equilibrated with TBS. IgG was then eluted using a two-step gradient: 50 mM Tris-HCl pH 7.5 containing 1M NaCl and 0.1 M Gly-HCl pH 2.6. The protein yield was monitored by the change in the optical density of the eluate at λ = 280 nm. Fractions of 1.5 mL were collected and dialyzed against 20 mM Tris-HCl pH 7.5.

### 4.5. ELISA of IgG1–IgG4 Subclasses

The relative concentration of IgG1–IgG4 was determined by ELISA. The assay kit contained two 96-well plates containing immobilized antibodies to IgG1–IgG4. The plate wells were incubated for 30 min at 37 °C with 100 μL IgG diluted with 50-fold serum diluent solution at a 0.1 mg/mL concentration. The plate wells were washed four times with 300 µL of phosphate-salt buffer. An amount of 100 µL of horseradish peroxidase conjugate against human IgG (Vector-Best, Russia, Novosibirsk) was added, incubated for 30 min at 37 °C, and washed four times with 300 µL of phosphate-salt buffer. A 100 µL solution of 3,3′,5,5′-tetramethylbenzidine was added and incubated for 10 min in darkness. The reaction was stopped by adding 100 µL of stop reagent (1.0 M H_2_SO_4_). Optical density was measured on a Multiskan FC spectrophotometer (Thermo Fischer Scientific, Waltham, MA, USA) in two-wavelength mode: the main filter of 450 nm and the reference filter of 620 nm. A calibration plot of optical density versus IgG1–IgG4 concentration was plotted in MS Excel. The concentration in the test samples was determine from the calibration graphs. The result was presented as the mean value in a series of three experiments.

### 4.6. ELISA of Anti-DNA-IgG

The content of IgG against double-stranded DNA (dsDNA-IgG) was determined by enzyme immunoassay using a kit A-8656 (Vector-Best, Russia, Novosibirsk) according to the manufacturer’s instructions. The kit contained control samples with known concentrations of anti-dsDNA antibodies: 0, 12.5, 50, 100, and 200 U/mL. Based on the optical density of the control samples and their concentration, a standard curve was plotted. The concentration of anti-dsDNA-IgG in samples was calculated from the optical density of standard samples, the results were presented as the mean values in a series of three independent experiments.

### 4.7. Analysis of DNase Activity

DNA hydrolysis activity was analyzed using supercoiled pBluescript DNA as previously described in [25,73,74]. An amount of 20 μL of reaction mixture contained 18 μg/mL (6.1 nM) of supercoiled pBluescript DNA, 5.0 mM MgCl_2_, 1.0 mM EDTA, 20.0 mM Tris-HCl pH 7.5, and IgG in a final concentration of 0.01 mg/mL. Samples were incubated for 48 h at 37° C. The relative amount of DNA in the supercoiled, linear, and relaxed plasmid bands was analyzed using ImageQuant v.5.2 (Molecular Dynamics, Los Angeles, CA, USA). The activity of IgG preparations was determined by the decrease in the percentage of dsDNA converted from the supercoiled form to relaxed and linear forms. Control samples did not contain IgGs. All measurements were carried out in linear regions of hydrolysis (15–40% of DNA hydrolysis), and the complete transition of the supercoiled plasmid to the hydrolyzed form was taken as 100% activity.

### 4.8. Statistical Analysis

Experimental results are presented as mean ± standard deviation (SD) or median [Q1, Q3]. At least three independent experiments were carried out for each sample. The measurement error did not exceed 5–7%. The data were evaluated for normality using Shapiro–Wilk test. Most variables did not fit the normality assumptions (*p* < 0.05). To analyze clinical data, we used Pearson’s chi-squared test (categorical variables) or One Way ANOVA with Tukey post hoc test (quantitative variables). The Wilcoxon–Mann–Whitney or Kruskal–Wallis One-Way ANOVA tests were used for non-normally distributed variables. Two-tailed *p* < 0.05 was considered statistically significant. Spearman’s rank correlation coefficients were calculated to assess the correlation between antibody titers. Statistical analysis was carried out using the Statistica 10 program (StatSoft. Inc., Tulsa, OK, USA). Graphs were plotted using Origin 2019 (OriginLab Corporation, Northampton, MA, USA).

## 5. Conclusions

The results of this paper indicate that the contents of IgG against RBD and S-protein in the blood of COVID-19 recovered patient and Sputnik V vaccinated persons are rather low, 1.1–1.4% for antibodies against RBD and 0.2–0.6% for other S-protein fragments, confirming the dominant role of RBD in the antibody spectrum against S-protein. The analysis has revealed very low levels of DNA hydrolysis by IgG from almost all patients and donors. The levels of anti-DNA antibodies and DNA-hydrolyzing antibodies were significantly lower comparing to such autoimmune pathologies as SLE or MS. None of recovered patients reported any autoimmune symptoms in their anamnesis and did not manifest them nearly two years after the tests. Our data speak in favor that the COVID-19 and Sputnik V vaccination do not induce or enhance autoimmune processes associated with the production of anti-DNA or DNA-hydrolyzing antibodies. In addition, the low DNase activity of IgG observed in patients who were vaccinated or recovered has been shown not to be associated with antibodies against the S-protein and its RBD fragment, indicating the absence of autoimmune pathology associated with the production of anti-DNA-IgG and DNA-hydrolyzing antibodies and confirming the safety of adenovirus vaccines against SARS-CoV-2. To our knowledge, this paper is the first work analyzing the DNA-hydrolyzing antibodies in COVID-19.

## Figures and Tables

**Table 1:** Characteristics of patients and donors in the study groups.

Characteristics	HTD, (*n* = 25)	MTD, (*n* = 25)	HTV, (*n* = 25)	NTD, (*n* = 25)	Statistics **
Males, % (*n*)	40% (10) *	24% (6)	40% (10)	28% (7)	N.S. (df = 3; *p* = 0.51)
Age, years Mean ± SD	45.5 ± 16.3	40.4 ± 10.9	42.8 ± 13.0	41.0 ± 10.2	N.S. (ANOVA: df = 3; F = 0.86; *p* = 0.427)
Age ranges:					
Up to 35, % (*n*)	24% (6)	32% (8)	36% (9)	40% (10)	N.S. (df = 12; *p* = 0.78)
36–45, % (*n*)	44% (11)	44% (11)	40% (10)	28% (7)	
46–55, % (*n*)	8% (2)	12% (3)	8% (2)	20% (5)	
56–65, % (*n*)	16% (4)	12% (3)	12% (3)	12% (3)	
Over 65, % (*n*)	8% (2)	0% (0)	4% (1)	0% (0)	
Mean time between onset of first symptoms and blood collection, weeks Mean ± SD	12.0 ± 6.0	11 ± 5	7 ± 2	0	ANOVA: df = 2; F = 6.65; *p* = 0.002 Tukey test: HTD vs. MTD *p* = 0.99 HTD vs. HTV *p* = 0.01 MTD vs. HTV *p* = 0.008
Mean PR value IgG against S-protein Mean ± SD	10.3 ± 4.1	5.1 ± 1.5	10.0 ± 3.7	0.4 ± 0.2	ANOVA: df = 3; F = 246; *p* = 6.3 × 10^−45^ Tukey test: HTD vs. MTD *p* < 0.00001 HTD vs. HTV *p* = 0.99 MTD vs. HTV *p* < 0.00001 NTD vs. HTD *p* < 0.00001 NTD vs. MTD *p* < 0.00001 NTD vs. HTV *p* < 0.00001

* The number of male patients is given; the rest were females; the average data are given for all 25 patients. ** The significance of the differences was calculated using Pearson’s chi-squared test (for gender and age ranges) or One Way ANOVA with Tukey post hoc test. A *p*-value > 0.05 was considered not significant (N.S.).

**Table 2:** Contents of IgG subclasses in the blood serum preparations and IgG preparations.

	Contents of IgG Subclasses			
	IgG1	IgG2	IgG3	IgG4
Average content in human serum [51]	67.0	22.0	7.0	4.0
Healthy donors [51]	36.8 ± 5.6	43.6 ± 9.3	11.3 ± 2.4	8.3 ± 3.1
HIV infection [25]	43.6 ± 9.5	38.3 ± 9.5	12.8 ± 2.0	5.3 ± 1.8
SLE [32]	70.8 ± 2.0	20.6 ± 3.0	6.7 ± 1.5	1.9 ± 1.0
MS [23]	22.0 ± 2.0	36.0 ± 3.5	12.3 ± 1.0	29.7 ± 3.0
COVID-19 HTD	58.4 ± 15.3	30.9 ± 15.5	4.6 ± 3.4	6.1 ± 3.0
COVID-19 MTD	56.1 ± 22.3	33.8 ± 17.1	4.2 ± 4.5	5.8 ± 3.8
Sputnik V vaccinated (HTV)	49.2 ± 14.4	39.2 ± 14.5	6.9 ± 2.5	4.7 ± 3.4
NTD	63.0 ± 16.0	24.9 ± 16.4	9.3 ± 1.6	8.3 ± 4.4

## Data Availability

Most of the relevant raw experimental results are given in the Supplementary section. Other empirical data that do not relate to the personal data of donors can be provided by request to Anna Timofeeva.

## Supplementary Materials

The supporting information can be downloaded at: https://www.mdpi.com/article/10.3390/ijms232213681/s1.
