# Supplementary material for: Natural IgG against S-Protein and RBD of SARS-CoV-2 Do Not Bind and Hydrolyze DNA and Are Not Autoimmune
Source: Int J Mol Sci. 2022 Nov 8;23(22):13681. doi: 10.3390/ijms232213681 (PMC9693483; doi:10.3390/ijms232213681)
Supplement: Supplementary file 1 [file ijms-23-13681-s001.zip › ijms-2011236-supplementary.pdf]

International Journal of
Molecular Sciences

MDPI

Supplementary data

Table S1. IgG subclass ratios in donors who recovered from COVID-19 and those vaccinated with Sputnik V. The data were obtained by ELISA. The mean values for three independent experiments are presented, with errors of measurement not exceeding 5%.

Donor ID	IgG1	IgG2	IgG3	IgG4
		**HTD**		
1020	44.8	48.0	3.9	3.2
1026	52.4	39.3	1.6	6.8
1116	46.3	47.3	2.7	3.7
1117	58.2	26.7	9.3	5.8
1156	48.3	31.6	9.3	10.8
1159	75.3	17.0	4.5	3.2
1164	83.7	6.3	1.1	9.0
Mean ± SD	58.4 ± 15.3	30.9 ± 15.5	4.6 ± 3.4	6.1 ± 3.0
Median [Q1; Q3];	52.4 [46.3; 75.3];	31.6 [17.0; 47.3];	3.9 [1.6; 9.3];	5.8 [3.2; 9.0];
Min–Max	44.8–83.7	6.3–48.0	1.1–9.3	3.2–10.8
		**MTD**		
1017	27.9	52.6	13.2	6.3
1033	33.0	56.3	6.5	4.2
1040	60.0	26.8	4.9	8.3
1048	47.8	39.2	1.7	11.3
1090	56.5	34.2	1.3	8.0
1091	86.4	11.9	0.7	1.0
1095	81.7	15.3	1.4	1.6
Mean ± SD	56.1 ± 22.3	33.8 ± 17.1	4.2 ± 4.5	5.8 ± 3.8
Median [Q1; Q3];	56.5 [33.0; 81.7];	34.2 [15.3; 52.6];	1.7 [1.3; 6.5];	6.3 [1.6; 8.3];
Min–Max	33.0–81.7	11.9–56.3	0.7–13.2	1.0–11.3
		**VTD**		
2011	35.5	56.2	5.6	2.8
2012	40.6	44.9	10.2	4.3
2013	42.7	43.5	9.7	4.2
2017	59.0	22.6	7.2	11.1
2018	66.7	27.2	4.8	1.4
2019	66.5	24.0	7.5	2.0
2020	33.6	55.8	3.6	7.1
Mean ± SD	49.2 ± 14.4	39.2 ± 14.5	6.9 ± 2.5	4.7 ± 3.4
Median [Q1; Q3];	42.7 [35.5; 66.5];	43.5 [24.0; 55.8];	7.2 [4.8; 9.7];	4.2 [2.0; 7.1];
Min–Max	33.6–66.7	22.6–56.2	3.6–10.2	1.4–11.1
		**NTD**		
1027	66.4	21.1	5.3	7.1
1034	52.4	30.8	3.9	12.9
1044	80.0	10.9	3.9	5.2
1050	51.6	36.6	6.5	5.3
1059	81.9	9.0	2.6	6.5
1061	69.6	12.2	2.2	16.0
1037	38.8	53.5	2.7	5.,0
Mean ± SD	63.0 ± 16.0	24.9 ± 16.4	9.3 ± 1.6	8.3 ± 4.4
Median [Q1; Q3];	66.4 [51.6; 80.0];	21.1 [10.9; 36.6];	3.9 [2.6; 5.3];	6.5 [5.2; 12.9];

Min–Max	38.8–81.9	9.0–53.5	2.2–6.5	5.0–16.0

Table S2. IgG1–IgG4 antibody subclass ratio in IgG fractions (total IgG, RBD-IgG, and S*-IgG) isolated from the blood plasma of three donor groups. The assay was performed by ELISA. The mean values of three independent experiments are given, the error did not exceed 5%.

	IgG, %	RBD-IgG, %	S*-IgG, %
		**HTD**	
IgG1	58.4	48.7	14.8
IgG2	30.9	42.6	79.6
IgG3	4.6	6.2	2.2
IgG4	6.1	2.5	3.4
		**MTD**	
IgG1	56..1	51.0	22.7
IgG2	33.8	44.9	72.4
IgG3	4.2	1.4	1.5
IgG4	5.8	2.7	3.4
		**HTV**	
IgG1	49.2	43.4	48.6
IgG2	39.2	48.0	42.7
IgG3	6.9	5.5	6.2
IgG4	4.7	3.1	2.5

In the case of all used individual preparations of IgGs, titers of antibodies against DNA, their average values for each of the groups are presented (IU/ml; Supplementary Table S3): HTD (10.9 ± 21.8**), MTD (**16.6 ± 34.0**), HTV (**20.7 ± 59.4**), NTD (**11.3 ± 26.8**). It should be noted that the sets of relative concentrations of anti-DNA-IgG were different in each of the four groups and did not correspond to the Gaussian distribution.**

Table S3. Analysis of DNA-hydrolyzing antibody activity and ELISA of anti-dsDNA-IgG in four groups of patients and donors.

HTD		MTD		HTV		NTD	
DNA hydrolysis, %	dsDNA-IgG, IU/ml	DNA hydrolysisDNA, %	dsDNA-IgG, IU/ml	DNA hydrolysisDNA, %	dsDNA-IgG, IU/ml	DNA hydrolysis, %	dsDNA-IgG, IU/ml
10.1	3.9	5.2	0.0	3.4	0.0	3.0	0
7.4	0.0	10.7	49.6	2.4	279.5	8.3	81.7
7.0	0.0	7.7	0.0	3.4	3.9	2.5	102.4
8.5	0.0	8.3	0.0	4.3	0.0	1.1	47.9
3.3	0.0	18.2	0.0	2.9	113.5	0.4	0.0
5.8	30.1	5.3	10.0	0.5	0.0	4.7	0.5
4.6	0.0	8.0	0.0	1.3	0.0	7.8	2.0
4.4	0.0	3.0	0.0	0.0	0.0	3.5	2.0
6.7	10.5	3.9	0.0	15.1	5.8	2.5	0.0
4.5	2.0	9.5	122.8	6.1	0.0	2.3	0.0
4.4	0.0	16.4	0.0	4.3	0.0	3.7	0.0
8.1	0.0	4.8	8.1	5.6	14.3	4.4	0.0
8.5	0.0	0.3	5.5	6.8	0.0	10.1	1.8
11.7	1.0	6.7	3.0	10.9	0.9	6.0	0.0
10.5	28.2	4.5	1.5	7.8	0.0	2.7	0.0

8.8	0.0	4.4	0.1	11.3	0.0	4.5	0.0
9.0	0.0	7.8	0.0	9.6	0.0	0.9	0.0
5.7	85.0	6.6	0.0	9.2	0.0	9.9	11.2
6.5	58.9	7.4	13.9	10.8	58.5	6.3	29.2
7.4	10.2	7.4	13.5	9.7	21.6	3.2	0.0
6.6	1.2	7.0	0.0	14.0	14.2	7.5	0.0
11.1	42.4	6.6	93.8	13.2	0.0	8.5	0.0
0.0	0.0	7.4	6.0	9.7	6.5	8.7	1.8
2.4	0.0	6.1	87.5	12.2	0.0	7.3	0.0
4.9	0.0	4.4	0.0	8.9	0.0	0.6	0.7
Mean ± SD							
6.7 ± 2.8	10.9 ± 21.8	7.1 ± 3.8	16.6 ± 34.0	7.3 ± 4.4	20.7 ± 59.4	4.8 ± 3.0	11.3 ± 26.8
Median [Q1; Q3]; Min–Max							
6.7 [4.6; 8.5]; 0–11.7	0 [0; 10.2]; 0–85.0	6.7 [4.8; 7.8]; 0.3–18.2	0.1 [0; 10.0]; 0–122.8	7.8 [3.4; 10.8]; 0–15.1	0 [0; 6.5]; 0–279.5	4.4 [2.5; 7.5]; 0.4–10.1	0 [0; 2.0]; 0-102.4

Figure S1.

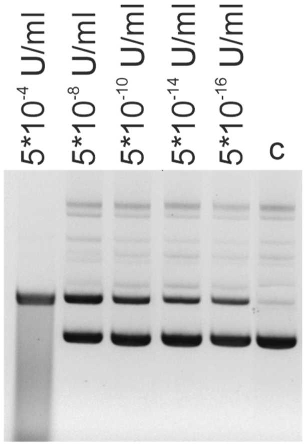

Figure S1. Hydrolysis of pBluescript plasmid DNA by DNase I at various final concentrations after incubation for 48 h at 37 °C; c – control samples incubated without enzymes. One unit of enzyme activity of DNase I, according to the supplier's instruction, completely degrades 1 μg of plasmid DNA in 10 min at 37 °C (EN0525, Thermo, Waltham MA, USA).

Table S4. Conversion of IgG catalytic activity to the enzyme activity units corresponding to the DNase I.

**HTD**		**MTD**		**HTV**		**NTD**	
Patient ID	DNase activity ×1019, U/mg of IgG	Patient ID	DNase activity ×1019, U/mg of IgG	Patient ID	DNase activity ×1019, U/mg of IgG	Donor ID	DNase activity ×1019, U/mg of IgG
1020	390	1017	39	2011	17	1021	57
1026	110	1033	520	2012	11	1027	8300
1116	92	1040	130	2013	17	1034	36
1117	190	1048	170	2017	26	1044	10
1156	16	1090	18000	2018	13	1050	5
1159	52	1091	41	2019	2,7	1059	280

1164	30	1095	150	2020	6,3	1061	5000
1167	27	1108	14	2021	3,4	1037	92
1168	80	1111	21	2022	4100	1038	36
1169	28	1129	300	2024	60	1039	30
1170	27	1150	7500	2025	26	1041	111
1131	150	1172	33	2026	48	1042	213
1132	190	1262	4	2027	84	1043	45000
1136	830	1168	80	2030	570	1051	960
1137	470	1169	28	2032	130	1056	43
1138	210	1170	27	2033	690	1058	230
1140	230	1171	130	2034	310	1062	8
1142	50	1173	76	2035	260	1067	37000
1143	73	1174	110	2036	550	1069	1300
1145	110	1175	110	2037	330	1096	69
1146	76	1182	92	2038	2400	1104	3900
1176	630	1184	76	2040	1700	1109	10000
1180	3,4	1189	110	2041	330	1114	12000
1231	11	1190	60	2042	1100	1119	3200
1241	34	1167	27	2062	220	1125	6
Median [Q1; Q3]; Min–Max x 10^19, U/mg	80 [30; 190]; 3.4–830		80 [33; 130]; 4.0–18000		130 [17; 550]; 2.7–4100		213 [36; 3900]; 5.0–45000
		N.A.[1]		N.A.		N.A.	

[1] N.A. - not applicable
